# The Psychosocial effects of the covid-19 lockdown`s on school-age childrens: A literature review

**DOI:** 10.1192/j.eurpsy.2023.1701

**Published:** 2023-07-19

**Authors:** O. A. Da Silva, H. Babani, G. D. O. Sarubi, A. M. S. Campos, A. L. S. Campos, J. G. A. Pontes, J. A. Ferreira

**Affiliations:** 1 School Of Medicine; 2 Centro Universitário Fametro, Manaus, Brazil

## Abstract

**Introduction:**

Home confinement was implemented worldwide as a response to the covid-19 pandemic. Therefore, almost all school-age children started to receive home-schooling from the beginning of 2020, it was necessary due to the length of the lockdowns. Being quarantined at home imposed an increase in psychological burden and the situation was aggravated because of school closure, lack of outdoor activity, aberrant dietary and sleeping habits, disrupting children’s usual lifestyle and promoting monotony, distress, impatience, annoyance, and varied neuropsychiatric manifestations.

**Objectives:**

This study aims to understand the correlation between quarantine and psychosocial effects on school-age children.

**Methods:**

An integrative literature review was developed in 3 steps: Development of the research question, search for scientific articles in the Pubmed database, and critical analysis of included articles. The search was conducted in September 2022, and articles between 2019 and 2022 were selected, for a total of 510 articles, of which 28 were used.

**Results:**

The confinement caused by the coronavirus imposed an immediate and lingering psychosocial impact on children due to drastic changes in their physical activity, lifestyle, and mental excursions. Even a short-term shutdown of educational institutions and home confinement is indeed troublesome and anticipated to have detrimental effects on children’s physical and mental health and shatter the sense of normalcy that schools used to provide. Another important factor to note is that some children`s had to be detached from their parents due to several factors, this juncture caused ever-lasting psychiatric consequences including post-traumatic stress disorder, anxiety, psychosis, depression, delinquency, and even suicidal tendency.

**Conclusions:**

Thus, frontline physicians must be aware of the psychosocial needs of the quarantined children. Hospital authorities need to make arrangements for children to communicate with parents via audiovisual devices. Government should invest in operational strategies to provide mental healthcare for the quarantined children.

**Disclosure of Interest:**

None Declared

